# Quality of written narrative feedback and reflection in a modified mini-clinical evaluation exercise: an observational study

**DOI:** 10.1186/1472-6920-12-97

**Published:** 2012-10-18

**Authors:** Elisabeth AM Pelgrim, Anneke WM Kramer, Henk GA Mokkink, Cees PM Van der Vleuten

**Affiliations:** 1 Department of Primary Care and Community Care, Radboud University Nijmegen Medical Centre, Postbus 9101 Huispostnummer 117, Nijmegen, HB, 6500, The Netherlands; 2 Department of Educational Development and Research, Faculty of Health, Medicine, and Life Sciences, Maastricht University, Maastricht, The Netherlands; 3 Radboud University Nijmegen Medical Centre, Nijmegen, The Netherlands; 4 King Saud University, Riyadh, Saudi Arabia; 5 University of Copenhagen, Copenhagen, Denmark

## Abstract

**Background:**

Research has shown that narrative feedback, (self) reflections and a plan to undertake and evaluate improvements are key factors for effective feedback on clinical performance. We investigated the quantity of narrative comments comprising feedback (by trainers), self-reflections (by trainees) and action plans (by trainer and trainee) entered on a mini-CEX form that was modified for use in general practice training and to encourage trainers and trainees to provide narrative comments. In view of the importance of specificity as an indicator of feedback quality, we additionally examined the specificity of the comments.

**Method:**

We collected and analysed modified mini-CEX forms completed by GP trainers and trainees. Since each trainee has the same trainer for the duration of one year, we used trainer-trainee pairs as the unit of analysis. We determined for all forms the frequency of the different types of narrative comments and rated their specificity on a three-point scale: specific, moderately specific, not specific. Specificity was compared between trainee-trainer pairs.

**Results:**

We collected 485 completed modified mini-CEX forms from 54 trainees (mean of 8.8 forms per trainee; range 1–23; SD 5.6). Trainer feedback was more frequently provided than trainee self-reflections, and action plans were very rare. The comments were generally specific, but showed large differences between trainee-trainer pairs.

**Conclusion:**

The frequency of self-reflection and action plans varied, all comments were generally specific and there were substantial and consistent differences between trainee-trainer pairs in the specificity of comments. We therefore conclude that feedback is not so much determined by the instrument as by the users. Interventions to improve the educational effects of the feedback procedure should therefore focus more on the users than on the instruments.

## Background

Research on formative assessment and feedback suggests that these are powerful tools to change trainees’ behaviour [1-4]. Formative assessment is an instructional intervention evaluating performance and identifying trainees’ strengths and weaknesses [1,5] in order to reveal performance gaps – i.e. differences between desired and actual performance [6]. From several studies we know that trainees do not benefit from feedback in the form of numerical marks [1,7,8], but that feedback should preferably be narrative and specific, explicating where more work needs to be done. Additionally, feedback can be made more effective when recipients receive guidance on how to turn feedback into concrete steps to improve their performance. Positive effects of narrative feedback have been reported by various authors, including Overeem et al. [9], who found higher satisfaction with such feedback, and Govaerts et al. [10], who suggested that narrative feedback can improve in-training evaluation. According to Sargeant et al. [11], feedback that is more specific is more readily assimilated, a view supported by Archer [12], who additionally concluded that feedback should not be exclusively trainer-driven but a two-way process in which trainers provide comments and at the same time encourage trainees to self-reflect on their performance. Archer’s model for effective feedback includes: *self-monitoring* (reflection on action) supported by *external feedback* and linkage *with personal goals* (action plan) in a coherent process rather than a series of unrelated events.

Since formative assessment of clinical performance often includes feedback provided by an expert trainer after direct observation of a trainee at work [13], several assessment instruments have been proposed to enhance the effectiveness of this type of feedback, mostly based on the mini-CEX [14,15]. Currently, a number of comparable instruments are widely used in workplace-based assessment. In order to determine the occurrence of self-assessment, recommendations by the trainer and explicit formulation of an action plan - elements resonating with Archer’s principles of *reflection, feedback* and *linking with personal goals*[12] -, Holmboe et al. [16] studied videotaped sessions in which supervisors provided oral feedback as part of a mini-CEX. Self-assessment *(reflection)* was found to be less frequent than recommendations made by supervisors *(feedback)*, while action plans *(linking with personal goals)* were rarely formulated. Based on these findings, we studied the effects on feedback of a modified mini-CEX. Like the original mini-CEX and similar assessment instruments [14], the instrument we studied is designed to generate feedback on observed performance during a clinical encounter. The instrument is tailored to practice settings in GP training in the Netherlands and the assessment form is designed to stimulate trainers and trainees to provide written narrative comments on trainee performance. We investigated the frequency of different types of comments invited in the form: self-reflection by the trainee, feedback from the trainer and an action plan proposed by both trainer and trainee. In view of the importance of the specificity of feedback [1,7,8,11,12], we also examined the specificity of the comments. We will use the word ‘feedback’ for written observations entered on the form by the trainer, ‘reflection’ for trainees’ written self-assessments and ‘action plan’ for written descriptions of learning goals, plans to achieve them and methods to evaluate the outcome. We use ‘comments’ with reference to all kinds of text entered by trainees and trainers on the form, including ‘feedback’, ‘reflections’ and ‘action plan’.

## Method

### Instrument

A modified mini-CEX was designed, including a form to evaluate trainees’ competence during an observed clinical encounter in general practice with additional space provided for answers to questions inviting trainers to provide narrative feedback and trainees to provide narrative reflections on ‘what went well’ and ‘what could have been done better’, and for an action plan drawn up by trainer and trainee, comprising learning goals, steps for improvement and ways of evaluating these. Additional file 1: Table S1 presents an English translation of the Dutch form. As our study focused on the written narrative comments, we did not analyse the quantitative components of the assessment form ( Additional file 1: Table S1), comprising different aspects of three competencies (medical expert, communicator and professional) and an overall judgement of competence on a 10-point scale, as is customary in Dutch education [17].

### Procedure and context

Within the postgraduate training programme in general practice in Nijmegen, the Netherlands, the above-described assessment form was introduced in March 2008 to stimulate GP trainers to give structured and systematic feedback on observed patient consultations conducted by GP trainees in the trainers’ practices. Every three months, at least three such assessments must be conducted. With regard to the written comments, the instructions for using the form state that, after an observed consultation the GP trainee first should give a short reflection on his/ her performance, followed by feedback from the trainer, after which trainer and trainee use the reflection and the feedback to draw up an action plan to address weaknesses.

*All trainers* attended a training programme of half a day each month and two days annually, dealing with *all aspects* of the work of a GP trainer, including assessment, of which the modified mini-CEX is a part. All trainers received a basic introduction about the modified mini-CEX assessment form. They watched a video of a patient consultation, assessed it using the form and discussed this with one another. During the other parts of the programme, trainers had opportunities to discuss and ask questions about observation and the use of the assessment form. *Trainees* were instructed about the overall assessment plan and the use of several assessment instruments (including the modified mini-CEX) at the beginning of their training. *Trainers and trainees* had permanent access to an online manual providing information about the relevance of observation and written narrative feedback for educational purposes and about the procedures.

### Participants and procedure

The above-described assessment form is in use during the postgraduate programme in general practice in Nijmegen, the Netherlands. During the first and last year of the three-year Dutch general practice programme, trainees work in a general practice, while training in the second year takes place in hospitals and other health care institutions. Since the assessment form is only used during the years in general practice, we studied the effects among first and third year trainees. Between March 2009 and September 2009 we asked GP trainees in Nijmegen who had started the first or third year of training in March of that year (N=69) to hand in their assessment forms. Since each trainee is supervised by one GP trainer for a whole year and each trainee is supervised by a different trainer, trainers and trainees were included in the study in pairs.

Participation was voluntary. Trainees were informed of the purpose of the study and they could voluntarily hand in their assessment forms at the institution in Nijmegen. They could make their forms anonymous by using a unique number to code them. At the time of the data collection, no ethical review board for medical educational research existed in the Netherlands. We fully complied with ethical rules in terms of voluntariness and anonymity. The researchers had no hierarchical relationship with either the trainees or the trainers

### Data analysis

We first calculated the percentage of forms with written comments in response to the seven requests for comments in the form (1: reflection, what went well, 2: reflection, what could have been done better, 3: feedback, what went well, 4: feedback, what could have been done better, 5: action plan, learning goals, 6: action plan, plan, 7: action plan, method of evaluation) ( Additional file 1: Table S1). Next, we rated the specificity of the comments on a three-point scale (specific, moderately specific, not specific). Feedback and reflection were rated as specific when it was clear to *which part* of the consultation they related, *what* did and did not go well and/or *why* it did or did not go well. An example of a specific comment relating to ‘what could have been done better’ is: ‘the consultation could have been finished more quickly’. A comment was rated as ‘moderately specific’ when it *only* indicated *which part* of the consultation did or did not go well or *what* did or did not go well or *why* a comment was made. An example of a moderately specific comment on ‘what went well’ or ‘what could have been done better’ is: ‘physical examination’. A comment was rated as ‘not specific’ when it was too general, relating to the consultation as a whole without specifying *which part* of the consultation was involved, *what* the comment referred to or *why* it was made. This type of very general unspecified comment – such as ‘pleasant contact’ - does not seem very useful, especially when it is read after a period of time has elapsed, because by then it will be difficult to recall which aspects of the trainee’s performance prompted the comment.

Comments on learning points were rated as specific if they explicitly stated *what* aspects needed more work. For example: ‘exploration of the differential diagnosis.’ A moderately specific comment is: ‘continue to think critically and logically’ and a ‘non-specific’ comment: ‘do more’. Comments about the planning were rated as specific if it was stated *how* the trainee could address a learning point. For example: ‘reminder on my desk’. A moderately specific comment is: ‘do not try to implement all learning points at once’ and a non specific comment: ‘practise’. Comments about the evaluation were rated as specific if it was stated *how progress would be monitored,* for example: ‘video recording’. A moderately specific comment on evaluation is: ‘mutual assessment’.

The criteria for specific, moderately specific and non specific comments were developed in a four step procedure. EP first read all the forms to gain an impression of how trainees had used the assessment form. Next, HM, AK and EP examined two forms (one with detailed and one with broad comments) and determined criteria for ‘specificity’, testing these criteria by independently rating five forms. After some small adjustments were made, the second version of the criteria was tested on 20 forms through independent rating by EP and AK (10 forms) and EP and HM (10 forms). The kappa coefficients for inter-coder agreement were .67 (EP/AK) and .77 (EP/HM). When agreement on coding was considered satisfactory, EP coded all the remaining forms. Discussion between EP and AK resolved uncertainty in regard of the rating for 30 of 485 forms due to poor legibility (14 forms) and doubts about categorisation (16 forms).

We used the data from trainees who handed in three or more forms to examine possible differences in specificity of comments of different trainee-trainer pairs. We calculated for each pair the percentage of specific comments, and we analysed differences (standard deviation) between pairs in the specificity of comments for each of the seven questions in the form. For this calculation we dichotomised the results in ‘specific comments’ and a second category containing all other comments and blank forms.

## Results

Of 485 forms returned by trainees, nine could not be related to an individual trainee, and the remaining 476 were from 54 different trainees, who had completed a mean number of 8.8 forms (SD 5.6; range 1–23). These trainees represented 78% of all the trainees invited to hand in their forms. Of the participating trainees, 68% were female and of the trainers 65% were male. These percentages are representative of the overall population of GP trainees and GP trainers in the Netherlands. The number of first year trainees exceeded that of third year trainees at the time of the study (57% were first year trainees). Also the first-year trainees returned more forms (78% first year trainees). Because of the between-group differences in return rate we examined whether there were quantitative differences between forms from first and third year trainees, but a chi-square analysis showed no significant differences (P > .05).

Table 1 shows the percentages of comments in response to the seven questions on the assessment form ( Additional file 1: Table S1), showing that reflection occurred less often than feedback and explicit formulation of an action plan was rare. Table 1 also shows the specificity of the comments for each of the seven questions. If comments were written down, the majority of the comments were specific (≥57%); and less than 10% was not specific.

**Table 1:** Numbers and percentages of forms with specific types of comments and the specificity of the comments

	**Forms with comments**		**Not specific %**	**Moderately specific %**	**Specific %**
	**N**	**(%)**			
Trainee self-reflection ‘what went well’	259	(53.4)	9.3	33.6	57.1
Trainee self-reflection ‘what could I have done better’	259	(53.4)	5.8	22.4	71.1
Trainer Feedback ‘what went well’	433	(91.3)	5.9	26.9	67.3
Trainer Feedback ‘what can be done better’	423	(87.2)	9.2	16.5	74.2
Action plan: ‘Learning goal’	166	(34.2)	5.5	20.8	74.0
Action plan: ‘Action plan’	57	(11.8)	6.0	16.4	77.6
Action plan: ‘Method of evaluation’	12	(2.5)	-	33.3	66.7

Because of the differences between trainee-trainer pairs in the number of completed forms (range 1–23), we wanted to explore possible differences in specificity between comments of different trainee-trainer pairs. To examine this, we used the forms of trainee-trainer pairs for which we had received at least three assessment forms. We calculated the mean percentage of specific comments per question per pair. Next we calculated the standard deviation (SD). Table 2 shows that SD’s are high, which means that there were large differences between pairs in the extent to which they formulated specific comments. Some pairs consistently provided specific comments on a certain question on all their assessment forms, while other pairs provided no specific comments relating to that question. This applies to all questions on the form, except for ‘evaluation’, which generally went unanswered.

**Table 2:** Percentages (SD) of specific comments by different trainee-trainer pairs (N=50) for each of the seven questions on the form

	**Mean percentage of specific comments (SD)**
Reflection: ‘what went well’	34.2 (29.7)
Reflection: ‘what could have been done better’	42.7 (28.5)
Feedback: ‘what went well’	62.5 (22.7)
Feedback: ‘what could have been done better’	67.5 (21.8)
Action plan: ‘Learning goals’	27.0 (27.8)
Action plan: ‘Plan’	11.2 (17.0)
Action plan: ‘Method of evaluation’	2.3 (8.1)

## Discussion

The results of this study show that the modified assessment form and procedure resulted in frequent reporting of feedback, less frequent reporting of reflection and only rare reporting of action plans. The results also show, however, that, generally, the reflections, feedback and action plans that were provided were specific. Based on the importance of specific comments as indicator of quality [1,7,8,11,12], we can conclude that, *if* comments were made, the modified assessment form elicited useful qualitative comments. It would be interesting, however, to further investigate the different frequencies of the different types of comments. Perhaps the modification, consisting only of encouragement and facilitation of written narrative reflections, feedback and an action plan, was not sufficiently powerful to induce trainers and trainees to make full use of the form. The way the assessment form was introduced and the availability of the online manual appear to have been inadequate to achieve reflective behaviour for all trainees and formulation of an action plan on a significant proportion of forms. Although the layout of the form directs which type of comment should be provided in which space, users retain the possibility to use it otherwise. It is also possible that feedback from the trainer and – to a lesser degree – reflection by the trainee are more firmly embedded in the assessment routine than linking these comments to the broader learning context by formulating an action plan. Perhaps, among trainers and trainees there already was a culture of giving feedback and, to a lesser extent, of reflecting on performance, but not (yet) of making plans for action to follow-up on feedback. This conclusion appears to be supported also by the large differences we found between trainee-trainer pairs. It seems that some pairs *do have* a culture of feedback, reflection and action plans, while for others such a culture remains to be developed. Apparently, some trainers and trainees do apply the information from assessment training and the online manual. These findings suggest that in order to enhance the effectiveness of assessment training, there should be a special focus on reflection and action plans. Additionally, trainers and trainees who use all the feedback modalities might be asked to share their experiences.

An important quality of our study is the response rate. A large number of assessment forms was analysed and almost 80% of GP trainees in the sample handed in their forms. However, the number of completed forms per trainee differed widely, with some trainees handing in only one form, even though the minimum required for the study period was six. The overrepresentation of forms from first year trainees may be attributable to the introduction of the new assessment form. Since the version of the form that was the subject of this study was first introduced in March 2008, first year trainees had used the form from their first day of training, while for the third year trainees (who had started their training before 2008) it meant a change. However, since the percentages of written comments in response to the seven questions did not differ between these groups, the overrepresentation of first year residents apparently did not impact on the results.

It should be noted, that although the reported action plans were specific, this finding is based on limited data, because the majority of forms did not contain an action plan. Only a few trainee-trainer pairs provided comments relating to an action plan. Next, we only studied written narrative comments entered in the assessment form. This is a limitation because we do not know what actually happened during the discussion between trainee and trainer when the text was formulated, and therefore a comparison with the results of Holmboe et al. cannot be made [16]. We chose our method because written narrative texts are one of the positive qualities of these formative assessment forms. Forms can be stored by trainees in their portfolio to help them reflect on a series of mini-CEX results in order to formulate learning goals, and they can help trainees and trainers to gain an overall impression of development of performance. Another limitation is the focus on the qualitative part of the assessment form. Further research should examine relationships between narrative feedback and the quantitative part of the assessment form.

In this study we looked at the written results of workplace-based observation and feedback. In a qualitative study [18] we examined ideas, barriers and motives experienced by trainers and trainees in relation to observation, reflection and feedback. Based on the results of the present study we would recommend a different approach to training to stimulate reflection in trainees and more attention to the formulation of an action plan, elements that are important for the effectiveness of feedback [12,19]. Further research is needed to explore how feedback and reflections that are specific as well as goal-oriented, as evidenced by the formulation of an action plan, impact on performance improvement, the ultimate aim of assessment of observed performance. The implications of the substantial differences between trainee-trainer pairs in relation to the percentages of specific comments require further investigation as well.

## Conclusion

The main findings of this study are that self-reflection by the trainee and formulation of an action plan were not uniformly reported on the assessment forms, the comments on the forms were generally specific and there were substantial, consistent differences between trainee-trainer pairs in the provision of specific comments. Based on these findings, we conclude that it is not so much the instrument (form and instructions) but rather the users that determine how the modified mini-CEX form is used. This suggests that interventions to improve the educational effectiveness of the feedback procedure should focus more on the users than on the instruments.

## Competing interest

The authors report no declarations of interest.

## Authors’ contribution

**EAMP** is a researcher at the Department of Primary Care and Community Care, Radboud University Nijmegen Medical Centre, The Netherlands. She is currently working on her PhD project on feedback based on direct observation of clinical encounters in workplace-based assessment in the GP-training setting. **AWMK**, PhD, is a senior researcher and leader of the research programme ‘profesional development in general practice’, at the Department of Primary Care and Community Care, Radboud University Nijmegen Medical Centre, The Netherlands. She also works as a general practitioner in Utrecht. **HGAM**, PhD, is methodologist at the Department of Primary Care and Community Care, Radboud University Nijmegen Medical Centre, The Netherlands. **CPMvdV**, PhD, is a Professor of Education, Chair of the Department of Educational Development and Research, Scientific Director of the School of Health Professions Education (SHE) at Maastricht University, The Netherlands. He holds honorary appointments in the University of Copenhagen (Denmark), King Saud University (Riyadh) and Radboud University (Nijmegen). All authors read and approved the final manuscript.

## Pre-publication history

The pre-publication history for this paper can be accessed here:

http://www.biomedcentral.com/1472-6920/12/97/prepub

## Supplementary Material

Additional file 1 **Table S1.** The modified mini-CEX: tailored to general practice and modified to explicitly encourage written self-reflection (trainee), feedback (trainer) and action plan (trainee and trainer). Click here for file

## References

[B1] Black P William D Assessment and classroom learning Assessment in Education 1998 5 7 74 10.1080/0969595980050102

[B2] Black P Wiliam D Inside the black box - raising standards through classroom assessment Phi Delta Kappan 1998 80 139 149

[B3] Norcini JB Burch V Workplace-based assessment as an educational tool: AMEE Guide No. 31 Medical Teacher 2007 29 855 871 10.1080/01421590701775453 18158655

[B4] Yorke M Formative assessment in higher education: moves towards theory and the enhancement of pedagogic practice High Educ 2003 45 477 501 10.1023/A:1023967026413

[B5] Krasne SW Wimmers PF Relan A Drake TA Differential effects of two types of formative assessment in predicting performance of first-year medical students Advanced in Health Sciences Education 2006 11 155 171 10.1007/s10459-005-5290-9 16729243

[B6] Rudolph JW Simon R Raemer DB Eppich WJ Debriefing as formative assessment: closing performance gaps in medical education Acad Emerg Med 2008 15 1010 1016 10.1111/j.1553-2712.2008.00248.x 18945231

[B7] Rolfe I Mcpherson J Formative assessment - how Am I doing Lancet 1995 345 837 839 10.1016/S0140-6736(95)92968-1 7898233

[B8] Shute VJ Focus on formative feedback Rev Educ Res 2008 78 153 189 10.3102/0034654307313795

[B9] Overeem K Lombarts MJ Arah OA Klazinga NS Grol RP Wollersheim HC Three methods of multi-source feedback compared: a plea for narrative comments and coworkers' perspectives Medical Teacher 2010 32 141 147 10.3109/01421590903144128 20163230

[B10] Govaerts MJ van der Vleuten CP Schuwirth LW Muijtjens AM The use of observational diaries in in-training evaluation: student perceptions Adv Health Sci Educ 2005 10 171 188 10.1007/s10459-005-0398-5 16193400

[B11] Sargeant J Mann K van der Vleuten C Metsemakers J "Directed" self-assessment: practice and feedback within a social context J Contin Educ Health Prof 2008 28 47 54 18366127 10.1002/chp.155

[B12] Archer JC State of the science in health professional education: effective feedback Medical Education 2010 44 101 108 10.1111/j.1365-2923.2009.03546.x 20078761

[B13] Watling CJ Lingard L Toward meaningful evaluation of medical trainees: the influence of participants' perceptions of the process Adv Health Sci Educ 2012 17 183 194 10.1007/s10459-010-9223-x 20143260

[B14] Pelgrim EA Kramer AW Mokkink HG van den Elsen L Grol RP van der Vleuten CP In-training assessment using direct observation of single-patient encounters: a literature review Adv Health Sci Educ 2011 16 131 142 10.1007/s10459-010-9235-6 PMC3074070 20559868

[B15] Kogan JR Holmboe ES Hauer KE Tools for direct observation and assessment of clinical skills of medical trainees: a systematic review JAMA 2009 302 1316 1326 10.1001/jama.2009.1365 19773567

[B16] Holmboe ES Yepes M Williams F Huot SJ Feedback and the mini clinical evaluation exercise J Gen Intern Med 2004 19 558 561 10.1111/j.1525-1497.2004.30134.x 15109324 PMC1492325

[B17] Ten Cate J Braak E Fenkel J Van der Pol A De 4-tot10 verwacht niveau-schaal (410VN-schaal) bij persoonlijke beoordelingen Tijdschrift voor Medisch Onderwijs 2006 25 31 10.1007/BF03056737

[B18] Pelgrim EAM Kramer AWM Mokkink HGA Van der Vleuten CPM The process of feedback in workplace-based assessment: organization, delivery, continuity Medical Education 2012 46 604 612 22626052 10.1111/j.1365-2923.2012.04266.x

[B19] Sargeant JM Mann KV van der Vleuten CP Metsemakers JF Reflection: a link between receiving and using assessment feedback Adv Health Sci Educ 2009 14 399 410 10.1007/s10459-008-9124-4 18528777

