# Supplementary material for: Quality of written narrative feedback and reflection in a modified mini-clinical evaluation exercise: an observational study
Source: BMC Med Educ. 2012 Oct 18;12:97. doi: 10.1186/1472-6920-12-97 (PMC3505168; doi:10.1186/1472-6920-12-97)
Supplement: Additional file 1 — Table S1. The modified mini-CEX: tailored to general practice and modified to explicitly encourage written self-reflection (trainee), feedback (trainer) and action plan (trainee and trainer). [file 1472-6920-12-97-S1.pdf]

Appendix 1. The adjusted mini-CEX tool.

Short Clinical Assessment

Name of GP-resident: **Name of GP-trainer**: **Date**:

Setting: consultation / telephone / home-call / duty / other, namely...

Patient: new / follow-up **complexity**: low / moderate / high

**1. MEDICAL EXPERT**	**≤4**	**5**	**6**	**7**	**8**	**9**	**10**	**not observed**

- Considers patients contextual circumstances (medical history, psychosocial)
- Asks relevant questions
- Performs relevant physical examination in a correct manner
- Makes a correct (preliminary) diagnosis
- Offers adequate treatment help
- Avoids superfluous medical care
- Logical sequencing of contact

**2. COMMUNICATOR**	**≤4**	**5**	**6**	**7**	**8**	**9**	**10**	**not observed**

- Explores request for help
- Explores cognitions, emotions and illness behaviour
- Connects problem analysis to the request for help
- Makes shared decisions with the patient about treatment
- Uses understandable language
- Explains within patient's frame of reference
- Empathizes
- Leads and keeps an eye on the (time)schedule

**3. PROFESSIONAL**	**≤4**	**5**	**6**	**7**	**8**	**9**	**10**	**not observed**

- Treats respectfully and accurate and is sensitive to shame
- Takes responsibility
- Keeps balance between involvement and distance

**4. OVERALL JUDGEMENT OF COMPETENCE**	**≤4**	**5**	**6**	**7**	**8**	**9**	**10**

**REFLECTION OF GP-RESIDENT**	**GP-TRAINERS' FEEDBACK**
What went well?	What went well?
What could be done better?	What could be done better?

ACTION PLAN *(specify: learning goals, plan, and method of evaluation)*
